# Developmental evaluation as a strategy to enhance the uptake and use of deprescribing guidelines: protocol for a multiple case study

**DOI:** 10.1186/s13012-015-0279-0

**Published:** 2015-06-18

**Authors:** James Conklin, Barbara Farrell, Natalie Ward, Lisa McCarthy, Hannah Irving, Lalitha Raman-Wilms

**Affiliations:** Department of Applied Human Sciences, Concordia University, Montreal, Quebec Canada; Bruyère Research Institute, Ottawa, Canada; Department of Family Medicine, University of Ottawa, Ottawa, Canada; School of Pharmacy, University of Waterloo, Waterloo, Ontario Canada; School of Sociological and Anthropological Studies, University of Ottawa, Ottawa, Canada; Leslie Dan Faculty of Pharmacy, University of Toronto, Toronto, Ontario Canada; Women’s College Research Institute, Toronto, Ontario Canada

**Keywords:** Polypharmacy, Overtreatment, Deprescribing, Developmental evaluation, Evidence-based guidelines

## Abstract

**Background:**

The use of developmental evaluation is increasing as a method for conducting implementation research. This paper describes the use of developmental evaluation to enhance an ongoing study. The study develops and implements evidence-based clinical guidelines for deprescribing medications in primary care and long-term care settings. A unique feature of our approach is our use of a rapid analytical technique.

**Methods/Design:**

The team will carry out two separate analytical processes: first, a rapid analytical process to provide timely feedback to the guideline development and implementation teams, followed by a meta-evaluation and second, a comprehensive qualitative analysis of data after the implementation of each guideline and a final cross-case analysis. Data will be gathered through interviews, through observational techniques leading to the creation of field notes and narrative reports, and through assembling team documents such as meeting minutes. Transcripts and documents will be anonymized and organized in NVIVO by case, by sector (primary care or long-term care), and by implementation site. A narrative case report, directed coding, and open coding steps will be followed. Clustering and theming will generate a model or action map reflecting the functioning of the participating social environments.

**Discussion:**

In this study, we will develop three deprescribing guidelines and will implement them in six sites (three family health teams and three long-term care homes), in a sequential iterative manner encompassing 18 implementation efforts. The processes of 11 distinct teams within four conceptual categories will be examined: a guideline priority-setting group, a guideline development methods committee, 3 guideline development teams, and 6 guideline implementation teams. Our methods will reveal the processes used to develop and implement the guidelines, the role and contribution of developmental evaluation in strengthening these processes, and the experience of six sites in implementing new evidence-based clinical guidelines. This research will generate new knowledge about team processes and the uptake and use of deprescribing guidelines in family health teams and long-term care homes, with a goal of addressing polypharmacy in Canada. Clinicians and researchers creating clinical guidelines to introduce improvements into daily practice may benefit from our developmental evaluation approach.

**Electronic supplementary material:**

The online version of this article (doi:10.1186/s13012-015-0279-0) contains supplementary material, which is available to authorized users.

## Background

The use of multiple medications (polypharmacy) by elderly patients is a significant problem in Canada. Fifty-three percent of seniors in health care institutions and 13 % living in the community surveyed take five or more medications, and this use increases with age [1]. Health administrative data shows that 59 % of seniors had claims for at least five drug classes in 2002, which increased to 66 % by 2012 [2].

Multiple and inappropriate medication use in the elderly can lead to non-adherence, medication errors, adverse drug reactions, fall risk, hospitalization, and mortality [3–11]. Screening tools can identify problematic medications [12, 13], and published algorithms can help clinicians to decide if medications should be stopped [14–16]. Small trials have evaluated the safety of stopping certain medications; however, no systematically developed guidelines have been published to help clinicians safely deprescribe (i.e., stop or decrease doses of medications causing problems or that are no longer needed).

We brought together four pharmacists, two physicians, and one social scientist to develop and implement three deprescribing guidelines in three family health teams (FHTs) and three long-term care (LTC) homes in Ontario. Guideline development will use the GRADE (Grading of Recommendations, Assessment, Development, and Evaluation) framework and AGREE (Appraisal of Guidelines, Research, and Evaluation) instrument. We are using two approaches to evaluate the implementation and uptake of these guidelines. First, we are using developmental evaluation (DE) to facilitate learning and improvement within our guideline development and implementation teams. Second, we are treating our development and implementation of three guidelines in six healthcare sites (representing 18 separate implementation efforts) as a natural laboratory for studying local strategies for implementing guidelines.

This paper explains our rationale for designing a project to create clinical guidelines, our use of DE to strengthen our process, and our use of a qualitative, multiple case study design to generate new knowledge about guideline implementation processes.

## The affordances and constraints of clinical guidelines

Clinical guidelines are both a popular and problematic way to introduce new approaches into frontline health care sites, though it is unclear how many physicians are actually using them [17–21]. Bell and colleagues (2013) estimate that there are “… at least 2400 guidelines in the Agency for Healthcare Research and Quality’s National Guideline Clearinghouse; more than 6400 guidelines in the database of the Guidelines International Network; and more than 2700 in the Canadian Medical Association’s CPG database” [17].

Several studies have explored barriers to guideline implementation. Cabana and colleagues [22] reviewed 76 studies and identified 293 barriers to physician use of guidelines, including unawareness of the guideline, unfamiliarity and disagreement with the recommendations, and low self-efficacy to implement recommendations. A Netherlands’ meta-review indicated that multi-faceted guideline implementation strategies are the most effective [23]. However, we are not yet able to predict which dissemination and implementation approaches are effective in different circumstances [24, 25].

Many studies have focused on specific instances of guideline implementation. These studies have identified hospital policies and management systems, leadership approaches, economic arrangements, time constraints, health care provider motivations, and patient motivations as barriers to guideline adoption [26–28]. Difficulties can also arise when opinions differ about the relative importance of using scientific evidence and clinical experience [29].

We have much to learn about guideline implementation [30, 31]. Some suggest that new approaches to knowledge translation might emphasize interaction processes rather than knowledge products such as clinical guidelines [32]. Gabbay and le May argue that clinicians rarely use guidelines and instead rely on collective knowledge sources that are embedded in the activities and artifacts of a practice [33, 34]. They use the term “mindlines” to refer to this fluid knowledge source, which is influenced by scientific findings, clinical judgment, organizational constraints, and ongoing interactions with peers. Lomas writes: “The approach to knowledge management in health services has generally been to try and deliver better-researched facts to clinicians and to try and help them to make good use of such facts. But this strategy assumes a rational and individualistic approach to knowledge acquisition that flies in the face of all the evidence about what some have called ‘the social life’ of knowledge—the intricate, convoluted and confusing pathways by which people in an organization negotiate, adapt and transform new knowledge that is often far from factual” [33].

Despite questions about the usefulness of guidelines, we opted to develop and implement three deprescribing guidelines in light of our clinical experiences and the paucity of literature in how to safely decrease or discontinue medications. Our project also attempts to pinpoint factors that influence attitudes and behaviors in complex social environments where guideline development and implementation occurs.

## Using developmental evaluation to address challenges related to innovations, programs, and interventions in complex environments

We use DE to identify effective and ineffective aspects of the guideline development and implementation processes and to bring findings to our guideline development and practice-site teams for their consideration. DE, with its complexity perspective, recognizes the importance of adapting programs to the circumstances of complex social environments [35–37]. When an intervention takes place under complex conditions, numerous factors interact with and influence each other, making it impossible to predict what will happen as the intervention moves forward. Patton suggests that such evaluations call for a focus on an interconnected web of relationships and influences [37].

A DE evaluator gathers data about the factors affecting a program’s functioning within a complex environment and helps innovators adapt to new circumstances [37, 38]. DE is useful when adapting a program to emerging conditions, modifying approaches for use in new contexts, developing scalable innovations, and generating feedback about an innovation as it moves forward [37, 39]. These uses are analogous to our development and testing of deprescribing guidelines.

Although a new method [37, 40], DE is being used by many health care researchers and evaluators. DE has been used to reveal program impacts [35], to facilitate social innovation through public health interventions [41], to reveal team dynamics that promote program development [42], to facilitate discussion of the impact of research [43], and to facilitate change through team dialog [44].

## Issues and challenges typical of DE

Three challenges have been identified by research teams using DE [41]. First, DE evaluators are interested in generating actionable knowledge to help their own team as opposed to generating knowledge applicable to other cases. Second, a DE evaluator is both a program team member and an evaluator of the team’s work, thus creating a significant cognitive burden. Third, the short timeframes needed for gathering and presenting DE data may differ from the longer timeframes needed for a thorough analysis of research data, thus delaying DE feedback sessions or rushing research analysis.

Our approach mitigates these risks in several ways. First, we will use separate analytical processes to meet our DE objectives and our research objectives. Our DE analysis is based on the qualitative technique of analytical memoing (described later in the paper) and will move forward in brief, rapid phases, while the full qualitative analysis is carried out in three increments after creation and implementation of each guideline.

We address the problem of the dual role by rotating the membership on our DE team, so several investigators and staff will have the experience of participating on guideline development teams and on the DE team. One principal investigator (the first author of this paper) will act as the DE team’s overall lead and will train new team members. All who participate in the DE work will create “bias statements” that identify potential conflicts that could arise; these are revisited and discussed at specific points during the project.

To strengthen our DE approach, we will conduct a meta-evaluation at three points during the project [45]. This involves considering our DE processes in terms of *The Program Evaluation Standards* [46]. This will require the DE team to reflect on and discuss issues concerning potential conflicts of interest, the adequacy of information sources, the appropriateness of data collection and analytical procedures, the extent of the evidence to support evaluation conclusions, and the impartiality of the feedback process.

## A design encompassing the goals of DE and translational research

We created eight research questions to contribute to an understanding of guideline development and implementation. These questions explore the use of consensus approaches to identify guideline topics and the impact of guidelines on clinician self-efficacy [see Additional File 1].

This paper describes our use of DE with the guideline development and implementation experiences of the six participating sites. The following research questions are relevant:

- What development and implementation processes can be used to create and introduce deprescribing guidelines into primary care and LTC contexts that positively influence the adoption and use of the practices described in the guideline?
- What are the barriers and facilitators to the use of deprescribing guidelines in primary care and LTC care contexts?
- What is the uptake and effect of deprescribing guidelines by health care professionals in primary care and LTC contexts?

To investigate these questions, we will develop and implement three deprescribing guidelines to assist prescribers in tapering and stopping medications that may no longer be needed or that can cause adverse effects in the elderly. The guidelines will be developed and implemented in a sequential, iterative manner. We begin by developing the first guideline and implementing it in three LTC facilities and three FHTs in Ontario. After this, we develop and implement the second and then the third guidelines.

As this work unfolds, we will gather data about our own processes to inform an ongoing DE process of learning and improvement to enhance the capacity of participating teams. We will also use the data to generate new knowledge about team processes and interactions that occur during the creation and implementation of clinical guidelines. The latter, which we refer to as our translational research subproject, relies on the same data as the DE process but uses more comprehensive analytical procedures.

This project is a multiple case study [47–49]. Each case involves the development of a deprescribing guideline and its implementation into six frontline sites. Each of the three guidelines will be a case. We chose qualitative methods as we intend to study in detail the processes for developing and implementing guidelines [50] as they occur in their natural settings [51]. Our purpose is to explore the phenomena and to generate a detailed description of these processes [52].

Our case reports will include separate sections that describe each site’s implementation experience, findings from each of the two sectors (FHTs and LTC) considered separately, and a comparison of the findings. The strength of our approach lies in its ability to capture aggregate data to inform the experience of the FHTs and LTC homes as a whole, while also allowing us to provide information at the individual practice level.

## Incorporating developmental evaluation into the design

**Fig. 1:**
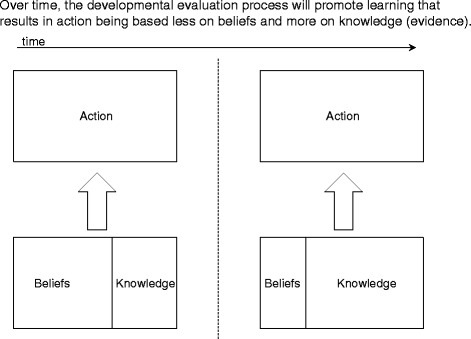
Evaluation learning framework for creating and implementing deprescribing guidelines

We devised a DE learning framework based on the idea that human actions are informed by intentions, and that human intentionality consists of beliefs (values, aspirations, assumptions) and knowledge (understanding of facts, usually based on evidence provided through experience) [37, 53]. Our DE process will reveal team member beliefs and knowledge, along with actions taken and results produced. These data will be fed into a deliberative process to allow team members to learn from their actions. This learning framework is illustrated in Fig. 1.

When the project begins, team members will have beliefs about how to implement the guidelines in the sites. As we work together, we will gather, analyze, and discuss data about our own development and implementation processes, which will allow us to identify and implement improvements.

## Data collection approach

**Fig. 2:**
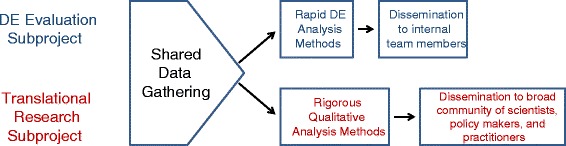
Shared data gathering and separate analytical methods

We created a single data collection approach to meet the needs of the DE evaluation and translational research subproject and separate analytical approaches for the DE evaluation and translational research (see Fig. 2).

The rest of this paper describes our research setting, data gathering approach, and analytical methods.

## Research setting

**Fig. 3:**
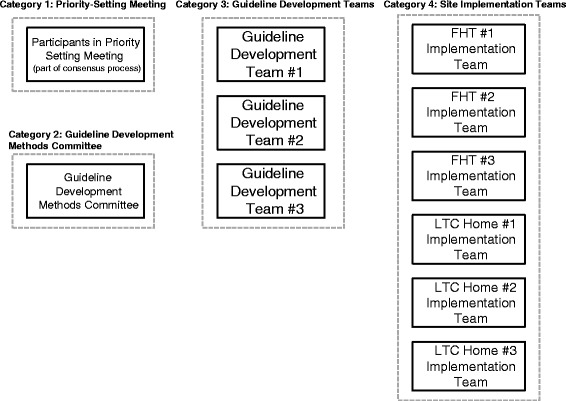
The research setting

Figure 3 depicts the research setting.

Our research setting includes 11 groups organized into four conceptual categories:

- A single group that participated in a priority-setting meeting as part of a process to select guideline topics
- A single group known as the guideline development methods committee (GDMC)
- Three guideline development teams (GDTs), each responsible for developing one guideline
- Six site implementation teams, three each from FHTs and LTC homes

The priority-setting meeting is part of a Delphi consensus process to identify medication classes for the three deprescribing guidelines. The Delphi method is a structured process for communicating and making decisions, which brings together a panel of experts who participate in several rounds of survey questionnaires. To design this process, team members came together for a priority-setting meeting to discuss interests and objectives, potential Delphi participants, and the survey design [54].

The GDMC consists of members of the investigator team with experience and/or interest in guideline development. This team determines and oversees the methods used by the three GDTs.

The research setting includes three GDTs established at different points during the project. Their role is to develop one evidence-based clinical guideline each. This work involves following the processes and standards determined by the GDMC, establishing roles and responsibilities, carrying out systematic reviews, reviewing the evidence, reaching agreement on guideline recommendations, creating, reviewing and revising a guideline template to present the results of the team’s work, presenting the guideline to the six implementation sites, and preparing articles for publication.

The research setting also includes the six sites that will implement the guidelines in their practices and provide us with data about their experiences. These Ontario FHTs and LTC homes were selected from interprofessional practices located in the Ottawa region that include a pharmacist.

## Data gathering methods

The project has four phases. Phases 1–3 focus on gathering and analyzing data related to each of the guidelines, while phase 4 involves a cross-case analysis.

### Guarding against confirmation bias

To guard against confirmation bias, team members who produce field notes will create a comprehensive record of the event that they are observing. Informal interactions are to be captured, along with discussions that pertain to agenda topics. Observers look for, and record, behaviors focused on the task at hand and the effectiveness of the team [55, 56]. Observers separate what they observe (behaviors and utterances) from interpretations. The field note template (see Additional file 2) encourages this distinction [50, 57, 58].

At least two team members will observe meetings and create field notes. Because researchers involved in data gathering have varied backgrounds (e.g., occupational therapy, psychology, pharmacy, medicine, social science), they will be subject to different occupational biases. We will use audio recordings to verify field note content.

Those who participate in gathering and analyzing data will create “bias statements” which summarize beliefs about deprescribing and guidelines. Team members with strong opinions about polypharmacy, the usefulness of evidence-based guidelines, or the most effective ways of implementing guidelines will identify and record those opinions in their bias statements. They will periodically review and reflect individually and collectively on how their views may influence how they collect, analyze, and interpret data.

### Phase 1 data collection

An overview of our data collection approaches for the various phases is presented in Additional file 3.

Data will be collected through observations resulting in field notes, as well as through narrative reports, interviews, and a review of relevant documents (such as meeting minutes). The activities that make up phase 1 are slightly different from those of subsequent phases, because phase 1 includes the consensus process to identify the topics of the three guidelines. For phase 1, we will gather data about the consensus process and about the development and implementation of the first guideline.

One investigator (JC) has primary responsibility for the data gathering and analysis methods used for the translational research and DE process. A method document will be created by this investigator and used to train those who participate in data gathering and analysis activities. This document explains when and how data is gathered and includes templates for field notes and interviews. Additional file 2 contains the field notes and one interview template, as examples.

During phase 1, a Delphi consensus process will be used to identify the topics for the three deprescribing guidelines [54]. The consensus process includes a priority-setting meeting to discuss design issues. Two team members will observe the meeting to produce field notes. Attendees will also produce brief narrative reports that document their meeting experience. These data will help us to consider how the consensus approach influences subsequent project activities.

We will seek to understand the functioning and influence of the GDMC by conducting semi-structured interviews with three committee members (a concluding interview with the same team members will be conducted in phase 3). All interviews conducted during the project will be digitally recorded, and a transcript will be created. We will also collect documents produced or used by this committee.

We will gather data about the first GDT by observing meetings and conducting interviews. Two observers will attend two meetings of the team and will produce field notes. We will conduct two semi-structured interviews of two team members (the team leader and coordinator), one at the beginning and one toward the end of the team’s process. We will also interview the two GDT members who carry out the systematic reviews that compile the evidence on which the guideline will be based. Finally, we will collect and review documents produced or used by the GDT.

Phase 1 includes capturing data about the processes used to implement the first guideline into the six sites. We will observe two implementation meetings at each site, yielding two field notes from each meeting (24 in total) and interview two implementation team members from each site; first, when the site is introduced to the guideline and the second approximately 3 months later, yielding 24 interview transcripts from implementation sites. We will interview 12 prescribers (two at each site) who used the guideline and will interview patients or family members from each participating site within 4 months of the intervention. We anticipate conducting two patient interviews for each site (12 in total). Hence, during phase 1 we will gather 24 field notes and 48 interview transcripts from the implementation sites.

Our dataset for phase 1 will thus include a minimum of 30 field notes and 57 interview transcripts.

### Phase 2 and phase 3 data collection

Data collection during phases 2 and 3 will be identical to phase 1, with these exceptions:

- The consensus process will be complete and thus no additional data will be gathered from that process
- In phase 2, we may collect relevant documents from the GDMC, and we will gather no interview data from the committee
- In phase 3, we may continue to collect relevant documents from the GDMC, and we will conduct semi-structured interviews with three committee members

Our dataset (aside from documents) for phase 2 will thus include a minimum of 28 field notes and 54 interview transcripts, and our dataset for phase 3 will include 28 field notes and 57 interview transcripts.

Additional file 3 contains a table summarizing our data gathering approach in the three phases.

## Analytical methods

We will use two analytical processes. The first will be a rapid process that allows the DE team to feedback data to the guideline development and implementation teams. This DE analysis includes a meta-evaluation that considers how the DE process could be improved. The second analytical process is a comprehensive investigation of patterns that characterize the participating teams over the course of the study. We will use NVIVO to organize data by case, by sector (FHT and LTC), and by participating site. We describe these two processes below.

### Rapid DE analytical process

The rapid DE analytical process ensures that lessons learned are available to the project teams in time for them to have an impact on team performance. Findings from the first guideline iteration will inform the second iteration, and findings from the second iteration will inform the third.

A team of DE analysts will review segments of the data to provide feedback to the guideline development and implementation teams. A minimum of three analysts will participate in each review. Each segment of data will focus on work that was conducted to develop or implement one of the guidelines. One segment will focus on the data gathered from the priority-setting meeting, a second segment will focus on the early work of the first guideline development team, a third will focus on the hand-over of the first guideline to the implementation sites, and so on.

The DE analysis team will produce two deliverables. The first will be analytical memos, produced by each member as they review the data. Memos will identify patterns evident in the data and insights that occur for the analyst as s/he reviews the data. Analytical memoing is a standard qualitative analysis technique for recording insights and interpretations as analytical procedures are implemented [50, 59]. Our team will use this technique to identify potential improvements in team functioning.

Completed memos will be distributed to members of the DE analysis team, who will read them, noting patterns and implications. The team then meets to create their second deliverable: a set of lessons learned to present to the guideline development and implementation teams. After the meeting, one analyst prepares a summary of the lessons. The feedback presentation will consist of lessons learned and a discussion of potential changes to team structures or processes.

### Meta-evaluation of the DE process

At the end of each guideline development and implementation cycle, three analysts will conduct a meta-evaluation of the work that has been done for that guideline. The meta-evaluation will be based on standards from *The Program Evaluation Standards* [49, 50] covering issues of utility, feasibility, ethics, and accuracy. Team members will individually read through a summary of the standards and make notes about how the standard applies to the work carried out in this iteration. The meta-evaluation team will then meet to share reflections and to discuss changes to the DE process. This meeting will yield a report that presents findings and proposed changes.

### Comprehensive qualitative analysis for the translational research subproject

The comprehensive analytical procedure includes a data review that produces a narrative case report, a directed coding step, and an open coding step. These procedures will be conducted by three team members and will generate three case study reports, one for the development and implementation of each guideline. The reports will provide a narrative account of the case, answers to the research questions, and an open-ended exploration of the data that might yield unanticipated discoveries [49, 60, 61]. They will also form the basis for the phase 4 cross-case analysis [48].

#### Data review and narrative case report writing

To conduct the data review, one analyst reads through all of the data. Because the case studies concern events that unfolded through time and in specific groups and locations, one analyst will review the data sequentially and in terms of the teams and sites from which the data was gathered. When reviewing the data from the implementation teams, the analyst will first look at the FHTs (starting with the first guideline) and then at the LTC sites (again starting with the first guideline). The analyst then writes a description of what happened during the development and implementation of the guideline. This descriptive story forms the first part of the case report. Two other analysts review the draft, after which a final draft is produced.

#### Directed coding

We will use a directed coding approach to generate answers to the research questions. The analyst will use a predetermined coding list developed from our research questions and DE framework to code portions of data [62, 63]. Additional file 4 presents our directed codes.

Directed coding will be carried out on the full qualitative data set, with the data grouped and coded in terms of the four categories that make up the research setting. Data from the guideline development teams will be coded as a self-contained segment, and data from each implementation site will be coded separately. This allows us to draw conclusions about each team, about the FHT and LTC sectors, and about the overall implementation experience.

This analysis is carried out by three team members. Two analysts will code approximately five identical pages of data. The full coding team then reviews the coded data, discussing and resolving discrepancies. One analyst then codes the rest of the data. When coding is complete, the coding team reviews and resolves issues and concerns. One analyst then reads through the coded data and creates a nuanced and complete statement in relation to that directed code (this could be an answer to one of the research questions or a statement concerning an aspect of the evaluation framework). Statements are reviewed and finalized before being added to the appropriate section of the case report.

#### Open coding

Open coding allows us to discover meaning in the data beyond that related to the research questions or evaluation framework. It allows the analyst to invent a classification scheme that emerges from the data [50, 62, 64].

Open coding will be performed on field notes, narrative reports, interview data, and documents. Analysts performing the open coding will distinguish between codes that apply to guideline development teams and to each FHT and LTC implementation teams. Codes will be defined to clearly indicate which context or group it relates to. If the meaning associated with one code also appears in other contexts or groups, additional codes can be created to capture these meanings. The open coding process will thus identify patterns that apply to a single implementation site or to multiple sites.

Open coding is conducted by three analysts. To begin, two analysts individually carry out open coding on five identical pages of data. The third analyst then joins them to review the coded data and pinpoint similarities and differences in codes that have been generated. At the end of this session, the participants agree on which codes to retain and which (if any) to discard. They also finalize the names and definitions of the codes.

One analyst then codes the rest of the data, noting issues or concerns that arise. As the coding proceeds, a second analyst will rejoin the effort on three occasions for one hour. During this time, the two analysts discuss and agree on codes for the section of data that is dealt with during the time frame, and they discuss issues that have arisen during the coding process.

The analyst who carried out the open coding then considers the strength of the codes by identifying how many times each code appears in the dataset and the number of data sources each code derives from. The analyst examines codes that appear infrequently, and in few data sources, and considers if any of these weak codes should be discarded. At this point, the three analysts review, discuss and resolve issues and concerns, and finalize the open codes.

#### Clustering and theming the open codes

Our clustering and theming procedure will reveal patterns that are evident in what participants say and do over the course of each iteration [52, 58, 60, 65–67]. It reveals patterns evident in their thinking (their intentions, beliefs, interpretations, and conclusions) and in the actions undertaken and results produced.

The clustering and theming process begins when open coding is complete and is consistent with procedures recommended by Braun and Clarke (2006) [67] and with the facilitation technique developed by the Institute for Cultural Affairs [65, 66]. Clustering will be done collaboratively by three analysts and includes a step to agree on a name, summary description, and detailed narrative description for each theme. Once themes have been agreed upon, the analysts consider how the patterns of thought and action revealed in the themes interact with each other to produce complex social environments that display an impulse toward improvement and change and a countering impulse toward stability and resistance. The final step will be to create a model to depict the functioning of these social environments [53, 68–70].

#### Cross-case analysis

The comprehensive qualitative analysis will be carried out once for each iteration of guideline development and implementation. The output from each round of analysis will be a case study report. Following this, we will conduct a cross-case analysis of the three case studies.

To do this, one analyst reviews the three case reports and writes summary descriptions of what the cases reveal about the functioning of guideline development and implementation teams; what changes and improvements are evident in the second and third guideline development and implementation processes; how patterns of thought, action, and structuring changed over the course of the three iterations; what results were achieved by the teams; what accounts for differences between the teams; what roles are evident among members of the participating teams; and what barriers and facilitators of development and implementation are evident in the three cases.

The analyst then compares the themes and models generated in the three cases and considers if some themes are evident across cases and whether changes in the thematic content of the cases suggest a process of improvement across iterations. The analyst also compares the models from the three cases.

The analyst concludes the cross-case analysis by comparing the answers to the research questions that were generated in the three cases. The analyst then creates a final answer for each research question based on an examination of the three cases, retaining important similarities and differences across the cases and offering explanations of differences. The analyst prepares a cross-case report to be distributed to the investigator team, which is finalized following agreed-upon revisions.

### Ethics approval

This protocol received ethics approval from the Bruyère Research Ethics Board (REB) (#M16-13-029); the University Human Research Ethics Committee (REC) at Concordia University (#30001626); the Ottawa Health Science Network REB (#20130589-10H); the Health Sciences REB at the University of Toronto (#29174); and the University of Waterloo REC (#19086).

## Discussion

Our research project seeks to strengthen the development and implementation of guidelines produced by our project teams while simultaneously studying the implementation processes used in six participating sites. We hope to offer recommendations to strengthen the relevance and uptake of research findings by incorporating reflective processes such as DE, action learning [71, 72], the plan-do-study-act cycle [73], or appreciative inquiry [74]. We also hope to build on the work of Anderson and colleagues [75] to further the understanding of barriers to and facilitators of developing and implementing deprescribing guidelines in primary care and LTC settings.

This research will contribute to our understanding of the ways in which DE might be incorporated into an implementation science research project and of the contribution that DE can make to a project’s knowledge translation goals. We will explore whether a rapid analytical procedure using analytical memos might overcome some challenges encountered by other researchers [41]. We will also discover whether guideline development teams and site implementation teams experience DE as having a positive (or negative) impact on their efforts to develop and implement deprescribing guidelines.

This research will generate new knowledge concerning the uptake and use of evidence-based deprescribing guidelines in frontline FHT and LTC sites. Recent research indicates that recommendations presented in evidence-based clinical guidelines are not integrated into practice through a simple, linear process but rather must penetrate complex ways of knowing and acting [33, 34]. Our research will follow the work of six implementation teams as they introduce deprescribing guidelines to clinicians and patients in their practice and will document the barriers and facilitators that they encounter. Our methods will allow us to identify experiences that are unique to a single site and those that are relevant for several sites.

Finally, the implementation science component of our project is nested within a broader project whose purpose is to ameliorate the serious problem of polypharmacy among Canada’s elderly. The rapid cycles of feedback enabled by DE allow for improvement of the processes through each iteration of guideline development and implementation. Ultimately, our project will produce guideline development and implementation methods that can be used for other medication classes, thus facilitating reach and impact of deprescribing guideline interventions with the aim of improving health in older persons.

## Additional files

Additional file 1:
**Complete list of research questions for the project.**
Additional file 2:
**Field note template and interview protocol.**
Additional file 3:
**Summary of data gathering activities and tools across the three phases.**
Additional file 4:
**Directed codes derived from the research questions and the evaluation framework.**

## Abbreviations

DE: developmental evaluation
FHT: family health team
GDT: guideline development team
LTC: long-term care
