# Supplementary material for: Developmental evaluation as a strategy to enhance the uptake and use of deprescribing guidelines: protocol for a multiple case study
Source: Implement Sci. 2015 Jun 18;10:91. doi: 10.1186/s13012-015-0279-0 (PMC4470007; doi:10.1186/s13012-015-0279-0)
Supplement: Additional file 1: — Complete list of research questions for the project. [file 13012_2015_279_MOESM1_ESM.doc]

# Additional File 1

**Complete List of Research Questions for the Project**

1. How can consensus approaches and available evidence be used by experts and stakeholders to identify priorities and create plans for deprescribing guidelines for use in primary care and long-term care contexts?

2. What development and implementation processes can be used to create and introduce deprescribing guidelines into primary care and long-term care contexts that positively influence the adoption and use of the practices described in the guideline?

3. What are the barriers and facilitators to the use of deprescribing guidelines in primary care and LTC care contexts?

4. What is the uptake and effect of deprescribing guidelines by health care professionals in primary care and long-term care contexts?

5. What is the projected cost-savings from the implementation of specific deprescribing guidelines?

6. Does the use of deprescribing guidelines improve clinician self-efficacy in tapering or stopping medications?

7. How do patients accept and feel about using deprescribing guidelines?

8. What research questions and potential evaluation frameworks regarding the impact of individual deprescribing guidelines can be generated?
