# Supplementary material for: Developmental evaluation as a strategy to enhance the uptake and use of deprescribing guidelines: protocol for a multiple case study
Source: Implement Sci. 2015 Jun 18;10:91. doi: 10.1186/s13012-015-0279-0 (PMC4470007; doi:10.1186/s13012-015-0279-0)
Supplement: Additional file 2: — Field note template and interview protocol. [file 13012_2015_279_MOESM2_ESM.doc]

# Additional File 2

**Field Note Template:**

Observer: _______________________________________________

Date and location: ________________________________________

| **Observations: what happened, in what order, things seen and heard** |
| --- |
| **Personal impressions, reactions, interpretations**  What patterns are evident in the way that group members interacted?  What barriers were encountered by group members?  What facilitated the work of the group?  [When Relevant] What is the uptake and effect of the deprescribing guideline?  [When Relevant] Are changes evident in provider self-efficacy? |
| **Reflections on the research design and methods** |

**Semi-Structured Interview Template for the initial interview with members of the Guideline Development Teams:**

1. What are your feelings towards guidelines in general?
2. How do you think the process of developing the guideline will go?
   1. What do you think will facilitate the process?
   2. What challenges do you anticipate?
3. What use do you see for a deprescribing guideline in primary care and long-term care?
