# Supplementary material for: Developmental evaluation as a strategy to enhance the uptake and use of deprescribing guidelines: protocol for a multiple case study
Source: Implement Sci. 2015 Jun 18;10:91. doi: 10.1186/s13012-015-0279-0 (PMC4470007; doi:10.1186/s13012-015-0279-0)
Supplement: Additional file 3: — Summary of data gathering activities and tools across the three phases. [file 13012_2015_279_MOESM3_ESM.doc]

# Additional File 3

Summary of Data Gathering Activities and Tools Across the Three Phases

| Phase 1 Activity | How Data is Gathered |
| --- | --- |
| Consensus process to identify guideline topics | - Observations at IT priority setting meeting (yielded two field notes) - Narrative reports produced by team members and subject matter experts who participated in priority setting meeting - Collection and review of documents produced or used by the consensus process team - Semi-structured interview of team member who coordinated the consensus process |
| First guideline development team process | - Observations at two guideline development team meetings, yielding two field notes from each of the two meetings - Semi-structured interviews of three members of the Guideline Management Committee, which sets and oversees the standards adopted by the team for the development of the guidelines [a concluding interview with these same team members will be conducted toward the end of Phase 3] - Two semi-structured interviews of two members of the first Guideline Development Team, one at the beginning of the team’s process and one toward the end of the team’s process (n=4) - Collection and review of documents produced or used by the GMC and by the first GDT - Semi-structured interviews of two members of the first GDT who were responsible for carrying out the systematic reviews |
| First guideline site implementation process | - Observations at two guideline implementation meetings at each of the six participating sites, yielding two field notes from each meeting, and 24 field notes in total - Semi-structured interviews of 12 Implementation Team members (two from each site) immediately after they are introduced to the guidelines and again three months later (n=24) - Semi-structured interviews of 12 prescribers (two at each site) who attempted to implement the guideline in their practice. Some of these prescribers were also implementation team members, and these questions were asked as part of the post-interview of implementation team members. - Interviews with up to five patients or family members from each participating site, scheduled to take place within 4 months of the intervention |
| Phase 2 & 3 Activity | How Data is Gathered |
| Second and third guideline development team process | - Observations at two guideline development team meetings in each phase, yielding two field notes from each meeting - Two semi-structured interviews of two members of the Phase 2 and 3 Guideline Development Teams, one at the beginning of the team’s process and one toward the end of the team’s process (n=4 for each phase) - Collection and review of documents produced or used by the Phase 1 and 2 GDTs - If appropriate: semi-structured interviews of members of the Phase 2 and 3 GDTs who were responsible for carrying out the systematic reviews or for creating the guideline content, to shed additional light on the team process - At the end of phase 3, semi-structured interviews of the same three members of the Guideline Management Committee who were interviewed in Phase 1 - Collection and review of documents produced or used by the GMT |
| Second and third guideline site implementation process | - Observations at two guideline implementation meetings at each of the six participating sites, yielding two field notes from each meeting, and 24 field notes in total for each phase - Semi-structured interviews of 12 Implementation Team members (two from each site) immediately after they are introduced to the guidelines and again three months later (n=24 for each phase) - Semi-structured interviews of 12 prescribers (two at each site) who attempted to implement the guideline in their practice. Some of these prescribers were also implementation team members, and these questions were asked as part of the post-interview of implementation team members. - Interviews with up to five patients or family members from each participating site, scheduled to take place within 4 months of the intervention |
