# Supplementary material for: Developmental evaluation as a strategy to enhance the uptake and use of deprescribing guidelines: protocol for a multiple case study
Source: Implement Sci. 2015 Jun 18;10:91. doi: 10.1186/s13012-015-0279-0 (PMC4470007; doi:10.1186/s13012-015-0279-0)
Supplement: Additional file 4: — Directed codes derived from the research questions and the evaluation framework. [file 13012_2015_279_MOESM4_ESM.doc]

# Additional File 4

Directed codes derived from the research questions

| **Research questions** | **Codes to use for the directed coding** |
| --- | --- |
| 1. How can consensus approaches and available evidence be used by experts and stakeholders to identify priorities and create plans for deprescribing guidelines for use in primary care and LTC caregiving contexts? | The full question will be used as the directed code. |
| 2. What development and implementation processes can be used to create and introduce deprescribing guidelines into primary care and LTC caregiving contexts that positively influence the adoption and use of the practices described in the guideline? | Break the question into two codes, to separate development from implementation processes:   - Development processes used to create and introduce deprescribing guidelines into primary care and LTC caregiving contexts that positively influence the adoption and use of the practices described in the guideline. - Implementation processes used to create and introduce deprescribing guidelines into primary care and LTC caregiving contexts that positively influence the adoption and use of the practices described in the guideline. |
| 3. What are the barriers and facilitators to the use of deprescribing guidelines in primary care and LTC caregiving contexts? | Break the question into two codes, to separate barriers from facilitators:   - Barriers to the use of deprescribing guidelines in primary care and LTC caregiving contexts. - Facilitators of the use of deprescribing guidelines in primary care and LTC caregiving contexts. |
| 4. What is the uptake and effect of deprescribing guidelines by health care professionals in primary care and LTC caregiving contexts? | The full question will be used as the directed code*.* |
| 6. Does the use of deprescribing guidelines improve prescriber/provider confidence in tapering or stopping medications? | The full question will be used as the directed code*.* |
| 7. How do patients accept and feel about using deprescribing guidelines? | Break the question into three codes, to separate positive, negative, and neutral reactions:   - Positive patient reactions to the use of deprescribing guidelines. - Negative patient reactions to the use of deprescribing guidelines. - Neutral patient reactions to the use of deprescribing guidelines. |

Directed codes derived from the evaluation framework

| **Research question and associated data** | **Codes to use for the directed coding** |
| --- | --- |
| 1. How can consensus approaches and available evidence be used by experts and stakeholders to identify priorities and create plans for deprescribing guidelines for use in primary care and LTC caregiving contexts?   - Observation field notes (from investigator meeting in July 2013, and any other relevant meetings) - Short narrative reports (written by the investigators after the July meeting) - Document Review (retrospective): Meeting minutes (and other documents) created and used by Delphi team. - Semi-structured interview of team member who coordinated the Delphi process. This person was involved in all Delphi steps. | Values, assumptions and beliefs about guidelines and guideline development  Values, assumptions and beliefs about implementing guidelines into practice environments  New learnings about guideline development and implementation derived from working on this team |
| 2. What development and implementation processes can be used to create and introduce deprescribing guidelines into primary care and LTC caregiving contexts that positively influence the adoption and use of the practices described in the guideline?   - Observation field notes of the three Guideline Development Team meetings and one teleconference after each Guideline Development Team meeting - Semi-structured pre and post interviews of Guideline Management Committee and Two Members of Each Development Team. - Document review (retrospective). - Semi-structured interviews of one or two people responsible for producing the content for each guideline. - Observation field notes of the Site Implementation Team meetings (initial meeting at which each guideline is introduced, followed by a second meeting at which use of each guideline is reviewed. - Semi-structured interviews of 12 Implementation Team members | Values, assumptions and beliefs about guidelines and guideline development  Values, assumptions and beliefs about implementing guidelines into practice environments  New learnings about guideline development and implementation derived from working on this team |
